# Supplementary material for: Promotional language and the adoption of innovative ideas in science
Source: Proc Natl Acad Sci U S A. 2024 Jun 11;121(25):e2320066121. doi: 10.1073/pnas.2320066121 (PMC11194578; doi:10.1073/pnas.2320066121)
Supplement: Supplementary file 1 — Appendix 01 (PDF) [file pnas.2320066121.sapp.pdf]

Promotional Language and the Adoption of Innovative Ideas in Science

(Supplementary Materials)

Hao Peng[1,2], Huilian Sophie Qiu[1,2], Henrik Barslund Fosse[3], and Brian Uzzi[1,2]

[1]Kellogg School of Management, Northwestern University, USA
[2]Northwestern Institute on Complex Systems, USA
[3]Novo Nordisk Foundation, Hellerup, Denmark

I. Data and Methods

A. Variables Used in the Regression Analysis

Table S1. This table lists our outcome variables, the key independent variable — the percentage of promotional words, and all predictor variables used in our regressions that have been found to influence grant evaluation in the literature. An "Y" indicates that the variable appears in the dataset. A "n/a" indicates that the variable is absent in the dataset.

Variable name	Definition and notes	NNF	NSF	NIH
Funded	Funding decision is coded as 1 if proposal was funded and 0 if was not funded.	Y	Y	Y
Innovativeness	Calculated using a widely used and validated novelty measure that characterizes whether a grant combines past knowledge in familiar or novel ways (1, 2).	Y	n/a	n/a
Citation Impact	Citation impact of publications based on an awarded grant is estimated using the journal impact factor (JIF) of the journal in which the paper is published. JIFs are published by The Web of Science. The publications of a grant are self-reported by the PI and checked by NNF.	Y	n/a	n/a
Productivity	Count of publications based on an awarded grant.	Y	n/a	n/a
% of promotional words	Percentage of the total number of occurrences of promotional words in a proposal's project description. The source and validation of our promotional words in this study is described in the main text.	Y	Y	Y
No. of words	Count of the total number of words in a proposal's project description.	Y	Y	Y
No. of references	Count of the number of references cited in a grant proposal.	Y	n/a	n/a
Concreteness score	A measure of how concrete the proposal is written. This variable is computed based on the grant's full text using the Brysbaert concreteness score (3).	Y	Y	Y
Flesch reading score	The grade-level needed to understand the grant's text. This variable is computed from the grant's full text using the Flesch reading ease measure (4); higher scores indicate clearer writing.	Y	Y	Y
Funding amount applied for	The amount of funding applied for (logged).	Y	Y	Y
Applicant's gender	Gender of application's PI (self-reported).	Y	Y	Y
Applicant's age group	Age group of application's PI (self-reported).	Y	n/a	n/a
No. of prior applications	Count of the number of previous applications submitted by the PI in each grant dataset at the time of each application.	Y	n/a	n/a
No. of prior grant successes	Same as above, but for the number of previously funded applications.	Y	n/a	n/a
No. of prior publications	Count of the number of publications of the PI up to the application year (collected by the grant agency).	Y	Y	Y
No. of prior citations	Same as above, but for citations. We measured this variable using quintiles because its distribution is skewed.	Y	Y	Y
Program area	Research fields measured as fixed effect.	Y	n/a	n/a
Instrument type	Types of grant applications measured as fixed effect.	Y	n/a	n/a
Application year	Year of application measured as fixed effect.	Y	Y	Y

B. Validation and Robustness Tests of Promotional Words

To further validate our promotional word lexicon and test the robustness of our findings, we conducted six additional robustness analyses. Below, we report details of four analyses (details of the other two tests are in the main text) that focus on (i) the construct validity of promotional words lexicon, (ii) the sensitivity of the overall dictionary, (iii) the sensitivity of the frequency in using the same promotional word in a proposal, and (iv) the context sensitivity of a promotional word.

(i) Independent Construct Validity Tests of the Promotional Word Dictionary. Millar and colleagues presented construct validity tests of their promotional dictionary in prior work (5, 6). We further tested construct validity by using the standard multi-trait, multimethod approach (MTMM) (7). According to the MTMM, if promotional words operate as purported by helping to communicate the originality, methods, and potential future directions of innovative ideas to readers (5), promotional words should correlate more strongly than their neutral synonyms with words that engender attention in readers. A word's valance and arousal levels have been shown to engender higher cognitive processing attention (8). Consequently, we should find that promotional words have higher valence and arousal levels than their neutral non-promotional synonyms (shown in Section V). For all 1,013 promotional-synonym word pairs (out of 1,506) that are indexed in an external valance and arousal lexicon (8, 9), we find that promotional words have statistically higher average valance and arousal scores than their synonyms using the weighted average, t-test, signrank, and mvtest of mean ($p < 0.002$ in all tests).

(ii) Promotional Word Lexicon Sensitivity. To address the concern of potential measurement errors in promotional words, i.e., some words might be invalid, we randomly removed promotional words from the lexicon at fraction of 5%, 10%, 15%, and 20%. For each percentage, we conducted 100 trials. In each trial, after shrinking the dictionary size randomly, we recounted the percentage of promotional words in each proposal and re-ran the regression to predict funding success. Across all 400 trails for each of the three datasets, the p-value of the main predictor variable, the percentage of promotional words, remains statistically significant ($p < 0.05$) except for one case out of 400 that occurred at 20% removal level in the NIH+NSF dataset. This test demonstrates the robustness of our analysis with regards to potential measurement error. Table S2 shows the statistics of the coefficients and p-values in all trials; the p-value is almost always below 0.01.

Table S2. Statistics of the coefficients and p-values of the main predictor variable, the percentage of promotional words in predicting funding acceptance. The coefficients and p-values (always below 0.02) are robust to different levels of potential measurement errors of the dictionary.

Dictionary words removed (%)	Regression Coefficients		p-values	
	Mean	Standard dev.	Mean	Standard dev.
**NNF**				
5%	39.3249	1.8826	2.4573e-10	5.8371e-10
10%	39.4990	2.8319	9.2506e-8	6.0084e-7
15%	40.9787	3.7637	7.8063e-8	4.4187e-7
20%	41.3978	4.5534	2.8179e-7	9.9244e-7
**NIH+NSF**				
5%	33.7934	1.9428	0.0099	0.0050
10%	34.8193	2.8725	0.0122	0.0075
15%	36.9854	3.6384	0.0111	0.0074
20%	37.0066	5.0355	0.0175	0.0148

(iii) Promotional Word Frequency Sensitivity. To address the concern that our main predictor variable, i.e., "the percentage of promotional words in a proposal" conflates the number of promotional words with the variety of promotional words in a proposal, we recounted only the first occurrence of each promotional word so that each unique promotional word is counted at most once per proposal. Then we re-ran the main regression with the new percentage variable to predict funding acceptance. The p-value in this robustness test and the p-value reported in the paper are almost identical ($p < 0.001$ for the NNF dataset; $p = 0.02$ for the NIH+NSF dataset).

(iv) Promotional Word Context Sensitivity. To address the context sensitivity of words in our dictionary, e.g., the promotional word "latest" can be used neutrally to indicate numerical ordering or promotionally to indicate originality, we conducted the following analysis. We first chose a removal rate of either 5%, 10%, 15%, or 20%. Then, for each occurrence of a promotional word in a proposal, we replaced it with a word randomly selected from the list of its neutral non-promotional synonyms plus the word itself, such that the word is non-deterministically replaced/discounted. The list of neutral synonyms and their validation is shown in Section V. The analysis showed that the regression coefficient of the percentage of promotional words in predicting funding success remains significant in 100 trails across all three datasets. Table S3 shows the statistics of the coefficients and p-values ($p < 0.001$ for the NNF dataset and is always below 0.05 for NIH+NSF dataset).

Table S3. Statistics of the coefficients and p-values of the main predictor variable, the percentage of promotional words in predicting funding acceptance. The coefficients remain stable and the p-values are all under 0.05 in the context-sensitivity tests.

Promotional words discounted (%)	Regression Coefficients		p-values	
	Mean	Standard dev.	Mean	Standard dev.
**NNF**				
5%	45.3835	0.1307	8.6656e-13	1.3499e-13
10%	45.4162	0.1818	9.0798e-13	1.9099e-13
15%	45.4285	0.2723	7.0450e-13	2.4914e-13
20%	45.4681	0.2839	1.0130e-12	3.2447e-13
**NIH+NSF**				
5%	22.5089	0.1511	0.0406	0.0013
10%	22.5304	0.1889	0.0407	0.0016
15%	22.6705	0.2705	0.0393	0.0022
20%	22.6030	0.3278	0.0407	0.0027

II. Regression Tables

This section provides full details of the regression models reported in the main text. In addition to the coefficients and statistics reported in the tables, we also conducted the following regression diagnostic tests: model fitness test using **BIC statistics, VIF, and Bonferroni correction** for multiple hypothesis test. Across all three tests, each of the models shows high robustness.

For all regression tables, the **BIC statistics** shows that adding our main predictive variable, the percentage of promotional words, significantly increases the explained variance of the model. For example, in Table S4 shown below, the BIC statistics drops more than 10 points from model 2 to model 3, which indicates that this predictor variable "significantly" increases the overall explanatory strength of the model (10).

Similarly, all values of the **VIF statistics** in each regression are below 10, except for the variable "No. Words" and the "Funding Amount Applied For". However, when we ran the same regression with the two variables omitted, the coefficients and statistical levels of the remaining variables did not meaningfully change. Specifically, the coefficient and standard error of our main predicator variable, the percentage of promotional words, remain highly significant at $p < 0.001$ and are similar to the reported values in each table. This suggests that, although the two variables have VIF statistics above 10, the ill-conditioning they may create when they are included in the model does not meaningfully change the reported results.

Lastly, we also ran the **Bonferroni correction** for multiple hypothesis testing and found that our main predictive variable, the percentage of promotional words, remains statistically significant at $p < 0.001$. The one exception to this test is that the logit model does not permit such a correction. Nevertheless, the very small *p*-value in the logit regression suggests that this variable would be robust to the Bonferroni correction because the *p*-value is so small to begin with.

In addition to the above statistical tests of the robustness of the regression models, **5-fold cross-validation of all regressions** indicated that the reported results were not due to overfitting. Furthermore, re-running the regressions with **Beta Coefficients** indicated that the percentage of promotional words had the second highest effect size among all variables in the regression, after the PI's prior grant application success (for DV = funding acceptance) or the PI's prior number of citations (for DV = avg/max JIF).

Table S4. Logit model predicting NNF grant funding decision (Y/N).

The percentage of promotional words in a grant predicts receiving funding after taking into account confounding variables. A “Y” indicates that the variable is included in the model. Significance levels: *** p<0.001, ** p<0.01, and * p<0.05. Standard errors are in the parentheses. The **VIF statistics and BIC statistics** show that the model is well specified and increases significantly in explained variance with the inclusion of promotional language.

	**Model 1**	**Model 2**	**Model 3**
**% of Promotional Words**	31.573*** (5.205)	32.063*** (5.374)	37.740*** (5.736)
No. Words: Quintile=1			0.213* (0.084)
No. Words: Quintile=2			0.456*** (0.084)
No. Words: Quintile=3			0.420*** (0.087)
No. Words: Quintile=4			0.752*** (0.113)
Concreteness Score			-0.048 (0.026)
Flesch Reading Score			-0.010 (0.021)
Funding Amount Applied For (log)			-0.070** (0.023)
PI Gender: Female		0.104* (0.052)	0.097 (0.052)
PI Age: 30-39		0.456 (0.236)	0.453 (0.238)
PI Age: 40-49		0.455 (0.237)	0.454 (0.241)
PI Age: 50-59		0.321 (0.240)	0.330 (0.244)
PI Age: 60-69		0.310 (0.247)	0.328 (0.250)
PI Age: >69		0.052 (0.326)	0.075 (0.329)
PI No. Prior Applications		-0.036** (0.013)	-0.034** (0.013)
PI No. Prior Grant Successes		0.340*** (0.035)	0.334*** (0.035)
PI No. Prior Publications		-0.001*** (0.000)	-0.001*** (0.000)
PI No. Citations: Quintile=1		0.054 (0.085)	0.065 (0.086)
PI No. Citations: Quintile=2		0.481*** (0.085)	0.487*** (0.085)
PI No. Citations: Quintile=3		0.744*** (0.087)	0.744*** (0.088)
PI No. Citations: Quintile=4		1.055*** (0.100)	1.050*** (0.100)
Submission Year (2015-2022)	Y	Y	Y
Instrument Type (5 categories)	Y	Y	Y
Program Area (3 categories)	Y	Y	Y
Constant	-1.649*** (0.091)	-2.415*** (0.255)	-1.366*(0.552)
Observations	13520	13520	13520

Table S5. Logit model predicting NNF grant funding decision (Y/N) (w/ novelty controlled).

The percentage of promotional words in a grant predicts receiving funding after taking into account confounding variables including novelty score. The number of observations drops from 13,520 to 11,550 because of missing bibliographic data for 1,970 grants. Statistical tests showed that the 13,520 and 11,550 samples did not statistically differ from each other. A "Y" indicates that the variable is included in the model. Significance levels: *** p<0.001, ** p<0.01, and * p<0.05. Standard errors are in parentheses. The **VIF statistics and BIC statistics** show that the model is well specified and increases significantly in explained variance with the inclusion of promotional language.

	**Model 1**	**Model 2**	**Model 3**
**% of Promotional Words**	28.743*** (5.696)	28.846*** (5.900)	33.989*** (6.363)
No. Words: Quintile=1			0.277** (0.094)
No. Words: Quintile=2			0.488*** (0.094)
No. Words: Quintile=3			0.438*** (0.099)
No. Words: Quintile=4			1.006*** (0.126)
**Innovativeness Score**			0.003 (0.002)
Concreteness Score			-0.056* (0.027)
Flesch Reading Score			0.004 (0.030)
Funding Amount Applied For (log)			-0.047 (0.026)
PI Gender: Female		0.110 (0.057)	0.101 (0.057)
PI Age: 30-39		0.428 (0.260)	0.459 (0.263)
PI Age: 40-49		0.385 (0.262)	0.425 (0.266)
PI Age: 50-59		0.246 (0.264)	0.297 (0.270)
PI Age: 60-69		0.251 (0.272)	0.301 (0.277)
PI Age: >69		0.003 (0.357)	0.069 (0.361)
PI No. Prior Applications		-0.027 (0.014)	-0.025 (0.014)
PI No. Prior Grant Successes		0.314*** (0.038)	0.312*** (0.038)
PI No. Prior Publications		-0.001** (0.000)	-0.001** (0.000)
PI No. Citations: Quintile=1		0.070 (0.094)	0.093 (0.095)
PI No. Citations: Quintile=2		0.520*** (0.093)	0.532*** (0.094)
PI No. Citations: Quintile=3		0.744*** (0.096)	0.749*** (0.097)
PI No. Citations: Quintile=4		1.085*** (0.109)	1.083*** (0.111)
Submission Year (2015-2022)	Y	Y	Y
Instrument Type (5 categories)	Y	Y	Y
Program Area (3 categories)	Y	Y	Y
Constant	-1.610*** (0.094)	-2.337*** (0.279)	-1.855** (0.610)
Observations	11550	11550	11550

Table S6. Logit model predicting NIH/NSF <u>grant funding decision (Y/N)</u>.

The percentage of promotional words in a grant predicts receiving funding after taking into account confounding variables. A "Y" indicates that the variable is included in the model. Significance levels: *** p<0.001, ** p<0.01, and * p<0.05. Standard errors are in parentheses. The VIF statistics and BIC statistics show that the model is well specified, increases significantly in explained variance with the inclusion of promotional language.

	**Model 1**	**Model 2**	**Model 3**
**% of Promotional Words**	27.644* (12.098)	28.998* (12.193)	29.541* (12.942)
No. Words: Quintile=1			-0.158 (0.150)
No. Words: Quintile=2			0.081 (0.165)
No. Words: Quintile=3			0.492** (0.181)
No. Words: Quintile=4			0.476* (0.196)
Concreteness Score			-0.054 (0.045)
Flesch Reading Score			0.176* (0.072)
Funding Amount Applied For (log)			-0.156*** (0.040)
PI Gender: Female		-0.034 (0.097)	-0.047 (0.098)
PI No. Prior Publications		-0.000 (0.000)	-0.000 (0.000)
PI No. Citations: Quintile=1		0.079 (0.142)	0.189 (0.146)
PI No. Citations: Quintile=2		0.102 (0.145)	0.226 (0.151)
PI No. Citations: Quintile=3		0.327* (0.150)	0.478** (0.157)
PI No. Citations: Quintile=4		0.466* (0.183)	0.576** (0.189)
Submission Year (2011-2020)	Y	Y	Y
Grant Name (2 categories)	Y	Y	Y
Constant	-0.678*** (0.170)	-0.900*** (0.206)	0.884 (0.553)
Observations	3210	3210	3210

Table S7. OLS model predicting the <u>innovativeness score</u> of NNF grants.

A higher percentage of promotional words in a grant predicts a higher innovativeness score after taking into account confounding variables. A "Y" indicates that the variable is included in the model. Significance levels: *** p<0.001, ** p<0.01, and * p<0.05. Standard errors are in parentheses. VIF statistics, BIC statistics, and a Bonferroni correction for multiple hypothesis show that the model is well specified, increases significantly in explained variance with the inclusion of promotional language, and is not biased by multiple hypothesis tests.

	**Model 1**	**Model 2**	**Model 3**
**% of Promotional Words**	188.060*** (25.064)	160.493*** (25.203)	139.686*** (26.434)
No. Words: Quintile=1			-0.517 (0.368)
No. Words: Quintile=2			0.099 (0.379)
No. Words: Quintile=3			0.039 (0.386)
No. Words: Quintile=4			-0.519 (0.473)
Concreteness Score			0.039 (0.118)
Flesch Reading Score			-0.438*** (0.125)
Funding Amount Applied For (log)			0.539*** (0.103)
**No. References**			0.050*** (0.010)
PI Gender: Female		-0.964*** (0.238)	-0.954*** (0.237)
PI Age: 30-39		-1.503 (1.012)	-0.491 (1.018)
PI Age: 40-49		-2.883** (1.017)	-1.664 (1.028)
PI Age: 50-59		-4.298*** (1.030)	-3.012** (1.042)
PI Age: 60-69		-2.897** (1.066)	-1.633 (1.077)
PI Age: >69		-3.941** (1.437)	-2.626 (1.442)
PI No. Prior Applications		0.196** (0.060)	0.191** (0.059)
PI No. Prior Grant Successes		0.130 (0.180)	0.140 (0.179)
PI No. Prior Publications		-0.005** (0.002)	-0.004** (0.002)
PI No. Citations: Quintile=1		0.829* (0.354)	0.896* (0.354)
PI No. Citations: Quintile=2		1.215*** (0.368)	1.149** (0.367)
PI No. Citations: Quintile=3		2.378*** (0.386)	2.238*** (0.386)
PI No. Citations: Quintile=4		2.714*** (0.452)	2.411*** (0.452)
Submission Year (2015-2022)	Y	Y	Y
Instrument Type (5 categories)	Y	Y	Y
Program Area (3 categories)	Y	Y	Y
Constant	9.599*** (0.428)	12.214*** (1.101)	-0.796 (2.424)
Observations	11550	11550	11550

Table S8. The OLS regression model predicting the average journal impact factor (JIF) of future publications supported by funded NNF grants.

The percentage of promotional words in funded grants predicts the average journal impact factor of their publications after taking into account confounding variables. A "Y" indicates that the variable is included in the model. Significance levels: *** p<0.001, ** p<0.01, and * p<0.05. Standard errors are in parentheses. VIF statistics, BIC statistics, and a Bonferroni correction for multiple hypothesis show that the model is well specified, increases significantly in explained variance with the inclusion of promotional language, and is not biased by multiple hypothesis tests.

	**Model 1**	**Model 2**	**Model 3**
**% of Promotional Words**	202.156*** (33.729)	185.972*** (33.478)	168.226*** (35.392)
No. Words: Quintile=1			-0.239 (0.492)
No. Words: Quintile=2			0.133 (0.490)
No. Words: Quintile=3			-0.451 (0.490)
No. Words: Quintile=4			-1.087 (0.661)
Concreteness Score			0.046 (0.146)
Flesch Reading Score			0.014 (0.144)
Funding Amount Granted (log)			0.673*** (0.136)
PI Gender: Female		-0.583 (0.318)	-0.519 (0.316)
PI Age: 30-39		-2.518 (1.583)	-1.176 (1.592)
PI Age: 40-49		-4.100* (1.591)	-2.652 (1.605)
PI Age: 50-59		-4.939** (1.604)	-3.439* (1.619)
PI Age: 60-69		-4.593** (1.652)	-3.088 (1.667)
PI Age: >69		-4.831* (2.028)	-3.386 (2.032)
PI No. Prior Applications		0.188 (0.098)	0.206* (0.098)
PI No. Prior Grant Successes		-0.070 (0.204)	0.006 (0.203)
PI No. Prior Publications		-0.009*** (0.002)	-0.008*** (0.002)
PI No. Citations: Quintile=1		0.821 (0.488)	0.973* (0.486)
PI No. Citations: Quintile=2		1.667** (0.515)	1.804*** (0.511)
PI No. Citations: Quintile=3		2.128*** (0.534)	2.280*** (0.532)
PI No. Citations: Quintile=4		3.472*** (0.637)	3.437*** (0.633)
Submission Year (2015-2022)	Y	Y	Y
Instrument Type (5 categories)	Y	Y	Y
Program Area (3 categories)	Y	Y	Y
Constant	4.744*** (0.497)	8.144*** (1.658)	-6.399 (3.345)
Observations	1137	1137	1137

Table S9. The OLS model predicting the max JIF of publications for funded NNF grants.

The percentage of promotional words in funded grants predicts the max journal impact factor of their publications after taking into account confounding variables. A "Y" indicates that the variable is included in the model. Significance levels: *** p<0.001, ** p<0.01, and * p<0.05. Standard errors are in parentheses. VIF statistics, BIC statistics, and a Bonferroni correction for multiple hypothesis show that the model is well specified, increases significantly in explained variance with the inclusion of promotional language, and is not biased by multiple hypothesis tests.

	Model 1	Model 2	Model 3
**% of Promotional Words**	433.486*** (80.778)	414.416*** (79.977)	366.586*** (83.869)
No. Words: Quintile=1			0.423 (1.166)
No. Words: Quintile=2			1.677 (1.161)
No. Words: Quintile=3			0.139 (1.161)
No. Words: Quintile=4			0.006 (1.566)
Concreteness Score			0.223 (0.347)
Flesch Reading Score			0.086 (0.341)
Funding Amount Granted (log)			1.940*** (0.322)
PI Gender: Female		-0.734 (0.759)	-0.534 (0.750)
PI Age: 30-39		-5.927 (3.782)	-1.764 (3.773)
PI Age: 40-49		-8.468* (3.802)	-3.912 (3.803)
PI Age: 50-59		-10.630** (3.831)	-5.873 (3.837)
PI Age: 60-69		-7.580 (3.947)	-2.769 (3.950)
PI Age: >69		-7.675 (4.845)	-3.195 (4.816)
PI No. Prior Applications		0.189 (0.235)	0.289 (0.233)
PI No. Prior Grant Successes		-0.018 (0.487)	0.190 (0.481)
PI No. Prior Publications		-0.015** (0.006)	-0.015** (0.005)
PI No. Citations: Quintile=1		1.978 (1.166)	2.518* (1.153)
PI No. Citations: Quintile=2		4.746*** (1.230)	5.134*** (1.211)
PI No. Citations: Quintile=3		5.340*** (1.277)	5.707*** (1.260)
PI No. Citations: Quintile=4		9.888*** (1.522)	9.749*** (1.500)
Submission Year (2015-2022)	Y	Y	Y
Instrument Type (5 categories)	Y	Y	Y
Program Area (3 categories)	Y	Y	Y
Constant	12.375*** (1.191)	18.234*** (3.962)	-25.073** (7.926)
Observations	1137	1137	1137

Table S10. The OLS model predicting the average and the max *field-normalized* journal impact factor (JIF) of publications based on funded NNF grants.

The percentage of promotional words in funded grants predicts the avg/max normalized JIF of their publications after taking into account confounding variables. The normalization computes the z-score associated with a journal's JIF percentile ranking among all journals in its corresponding discipline designated in the UCSD map of science catalog (11). The number of observations drops to 1,050 (from 1,137) due to missing discipline information for some journals. A "Y" indicates that the variable is included in the model. Significance levels: *** p<0.001, ** p<0.01, and * p<0.05. Standard errors are in parentheses. VIF statistics, BIC statistics, and a Bonferroni correction for multiple hypothesis show that the model is well specified, increases significantly in explained variance with the inclusion of promotional language, and is not biased by multiple hypothesis tests.

	**Model 3**<br>**DV = Avg. JIF (Z-score)**	**Model 3**<br>**DV = Max JIF (Z-score)**
**% of Promotional Words**	11.658** (4.260)	19.503*** (5.036)
No. Words: Quintile=1	-0.074 (0.059)	-0.018 (0.070)
No. Words: Quintile=2	-0.021 (0.059)	0.110 (0.069)
No. Words: Quintile=3	-0.117* (0.059)	-0.078 (0.069)
No. Words: Quintile=4	-0.249** (0.079)	-0.220* (0.094)
Concreteness Score	0.029 (0.018)	0.037 (0.021)
Flesch Reading Score	-0.002 (0.018)	-0.014 (0.021)
Funding Amount Granted (log)	0.089*** (0.016)	0.144*** (0.019)
PI Gender: Female	-0.100** (0.038)	-0.103* (0.045)
PI Age: 30-39	-0.163 (0.185)	-0.225 (0.219)
PI Age: 40-49	-0.307 (0.187)	-0.309 (0.221)
PI Age: 50-59	-0.415* (0.189)	-0.377 (0.223)
PI Age: 60-69	-0.395* (0.194)	-0.369 (0.230)
PI Age: >69	-0.453 (0.239)	-0.209 (0.282)
PI No. Prior Applications	0.029* (0.012)	0.020 (0.014)
PI No. Prior Grant Successes	0.020 (0.025)	0.060* (0.029)
PI No. Prior Publications	-0.001*** (0.000)	-0.001 (0.000)
PI No. Citations: Quintile=1	0.156** (0.059)	0.159* (0.070)
PI No. Citations: Quintile=2	0.234*** (0.062)	0.346*** (0.073)
PI No. Citations: Quintile=3	0.273*** (0.064)	0.374*** (0.076)
PI No. Citations: Quintile=4	0.439*** (0.076)	0.497*** (0.090)
Submission Year (2015-2022)	Y	Y
Instrument Type (5 categories)	Y	Y
Program Area (3 categories)	Y	Y
Constant	-0.537 (0.400)	-1.179* (0.473)
Observations	1050	1050

Table S11. Negative Binomial Model predicting the productivity of funded NNF grants.

The percentage of promotional words in funded grants statistically but weakly predicts their number of publications after taking into account control variables. A "Y" indicates that the variable is included in the model. Significance levels: *** p<0.001, ** p<0.01, and * p<0.05. Standard errors are in parentheses. VIF statistics, BIC statistics, and a Bonferroni correction for multiple hypothesis show that the model is well specified and increases significantly in explained variance with the inclusion of promotional language and is not biased by multiple hypothesis tests.

	**Model 1**	**Model 2**	**Model 3**
**% of Promotional Words**	17.920** (6.696)	18.935** (6.494)	15.723* (6.731)
No. Words: Quintile=1			0.129 (0.093)
No. Words: Quintile=2			0.316*** (0.092)
No. Words: Quintile=3			0.149 (0.092)
No. Words: Quintile=4			0.041 (0.121)
Concreteness Score			0.005 (0.028)
Flesch Reading Score			-0.022 (0.036)
Funding Amount Granted (log)			0.144*** (0.025)
PI Gender: Female		-0.020 (0.060)	-0.015 (0.059)
PI Age: 30-39		-0.605* (0.292)	-0.313 (0.290)
PI Age: 40-49		-0.587* (0.295)	-0.240 (0.294)
PI Age: 50-59		-0.609* (0.298)	-0.230 (0.297)
PI Age: 60-69		-0.734* (0.307)	-0.377 (0.306)
PI Age: >69		-0.615 (0.371)	-0.261 (0.367)
PI No. Prior Applications		-0.051** (0.019)	-0.042* (0.019)
PI No. Prior Grant Successes		0.085* (0.040)	0.106** (0.040)
PI No. Prior Publications		0.002*** (0.000)	0.002*** (0.000)
PI No. Citations: Quintile=1		-0.081 (0.095)	-0.031 (0.093)
PI No. Citations: Quintile=2		0.179 (0.098)	0.240* (0.096)
PI No. Citations: Quintile=3		0.180 (0.102)	0.220* (0.099)
PI No. Citations: Quintile=4		0.393*** (0.119)	0.382*** (0.116)
Submission Year (2015-2022)	Y	Y	Y
Instrument Type (5 categories)	Y	Y	Y
Program Area (3 categories)	Y	Y	Y
Constant	2.285*** (0.096)	2.674*** (0.310)	-0.749 (0.617)
lnalpha	-0.326*** (0.047)	-0.463*** (0.049)	-0.517*** (0.050)
Observations	1137	1137	1137

Table S12. The OLS and Negative Binomial Regression models predicting the Avg JIF, Max JIF, and Productivity for funded NNF grants, with control for a grant's novelty score.

The percentage of promotional words in funded grants predicts the citation impact and productivity after taking into account controls including the novelty score. A "Y" indicates that the variable is included in the model. Significance levels: *** p<0.001, ** p<0.01, and * p<0.05. Standard errors are in parentheses. VIF statistics, BIC statistics, and a Bonferroni correction for multiple hypothesis show that the model is well specified, increases significantly in explained variance with the inclusion of promotional language, and is not biased by multiple hypothesis tests.

	**Model 3**<br>**DV = Avg. JIF**	**Model 3**<br>**DV = Max JIF**	**Model 3**<br>**DV = No. Pubs**
**% of Promotional Words**	149.774*** (38.071)	337.067*** (89.918)	15.598* (7.095)
No. Words: Quintile=1	-0.107 (0.547)	0.421 (1.293)	0.029 (0.102)
No. Words: Quintile=2	0.420 (0.543)	1.826 (1.282)	0.227* (0.099)
No. Words: Quintile=3	-0.462 (0.544)	0.033 (1.286)	0.080 (0.100)
No. Words: Quintile=4	-1.238 (0.722)	-0.512 (1.706)	-0.027 (0.129)
**Innovativeness Score**	0.025* (0.012)	0.048 (0.029)	0.002 (0.002)
Concreteness Score	0.072 (0.152)	0.262 (0.358)	0.007 (0.028)
Flesch Reading Score	0.086 (0.151)	0.244 (0.356)	-0.010 (0.034)
Funding Amount Granted (log)	0.662*** (0.148)	1.851*** (0.350)	0.147*** (0.027)
PI Gender: Female	-0.336 (0.348)	-0.400 (0.823)	-0.072 (0.065)
PI Age: 30-39	-0.548 (1.626)	-0.678 (3.840)	-0.220 (0.293)
PI Age: 40-49	-2.372 (1.641)	-3.629 (3.875)	-0.148 (0.297)
PI Age: 50-59	-3.090 (1.658)	-5.295 (3.916)	-0.087 (0.301)
PI Age: 60-69	-2.777 (1.710)	-2.231 (4.038)	-0.274 (0.310)
PI Age: >69	-2.785 (2.129)	-1.822 (5.028)	-0.063 (0.379)
PI No. Prior Applications	0.244* (0.104)	0.305 (0.245)	-0.049* (0.020)
PI No. Prior Grant Successes	-0.130 (0.220)	0.017 (0.519)	0.121** (0.042)
PI No. Prior Publications	-0.009*** (0.002)	-0.015** (0.006)	0.002*** (0.000)
PI No. Citations: Quintile=1	1.011 (0.541)	2.545* (1.278)	-0.025 (0.102)
PI No. Citations: Quintile=2	1.620** (0.554)	4.618*** (1.310)	0.245* (0.103)
PI No. Citations: Quintile=3	2.629*** (0.589)	6.322*** (1.391)	0.157 (0.109)
PI No. Citations: Quintile=4	3.643*** (0.695)	10.089*** (1.640)	0.276* (0.126)
Submission Year (2015-2022)	Y	Y	Y
Instrument Type (5 categories)	Y	Y	Y
Program Area (3 categories)	Y	Y	Y
Constant	-8.648* (3.702)	-30.518*** (8.743)	-1.264 (0.677)
lnalpha			-0.526*** (0.054)
Observations	986	986	986

Table S13. The Logit model predicting NNF grant funding decision (Y/N) with or without control for the average sentiment score of a grant proposal.

The logit model that regresses funding success on a NNF grant's average sentiment score, the percentage of promotional words, and controls. Promotional words continue to predict funding acceptance after taking into account the positive effect of sentiment score. A "Y" indicates that the variable is included in the model. Significance levels: *** p<0.001, ** p<0.01, * p<0.05. Standard errors are in the parentheses.

	Model 1	Model 2	Model 3
**% of Promotional Words**		37.74*** (5.736)	31.46*** (6.790)
**Average Sentiment Score**	2.412*** (0.484)		1.001 (0.575)
No. Words: Quintile=1	0.217* (0.084)	0.213* (0.084)	0.218** (0.084)
No. Words: Quintile=2	0.472*** (0.085)	0.456*** (0.084)	0.464*** (0.085)
No. Words: Quintile=3	0.419*** (0.087)	0.420*** (0.087)	0.428*** (0.087)
No. Words: Quintile=4	0.769*** (0.114)	0.752*** (0.113)	0.771*** (0.114)
Concreteness Score	-0.0212 (0.025)	-0.048 (0.026)	-0.0490 (0.026)
Flesch Reading Score	-0.00966 (0.022)	-0.010 (0.021)	-0.00821 (0.022)
Funding Amount Applied For (log)	-0.0654** (0.024)	-0.070** (0.023)	-0.0710** (0.024)
PI Gender: Female	0.102 (0.0520)	0.097 (0.052)	0.103* (0.0521)
PI Age: 30-39	0.469* (0.238)	0.453 (0.238)	0.464 (0.238)
PI Age: 40-49	0.473* (0.241)	0.454 (0.241)	0.473* (0.241)
PI Age: 50-59	0.347 (0.244)	0.330 (0.244)	0.352 (0.244)
PI Age: 60-69	0.336 (0.250)	0.328 (0.250)	0.351 (0.251)
PI Age: >69	0.0740 (0.329)	0.075 (0.329)	0.101 (0.329)
PI No. Prior Applications	-0.034** (0.013)	-0.034** (0.013)	-0.034** (0.0123)
PI No. Prior Grant Successes	0.336*** (0.034)	0.334*** (0.035)	0.336*** (0.034)
PI No. Prior Publications	-0.001*** (0.000)	-0.001*** (0.000)	-0.001*** (0.000)
PI No. Citations: Quintile=1	0.0561 (0.0856)	0.065 (0.086)	0.0633 (0.0857)
PI No. Citations: Quintile=2	0.476*** (0.085)	0.487*** (0.085)	0.484*** (0.085)
PI No. Citations: Quintile=3	0.743*** (0.088)	0.744*** (0.088)	0.742*** (0.088)
PI No. Citations: Quintile=4	1.059*** (0.100)	1.050*** (0.100)	1.050*** (0.100)
Submission Year (2015-2022)	Y	Y	Y
Instrument Type (5 categories)	Y	Y	Y
Program Area (3 categories)	Y	Y	Y
Constant	-1.406* (0.555)	-1.366* (0.552)	-1.421* (0.553)
BIC (lower score indicates a better fit)	12025.07	12006.88	12013.39
Observations	13520	13520	13520

III. Consistency of Promotional Words Used in NIH, NSF, and NNF Proposals

The following bar charts show the top 30 highest frequency promotional words appearing in each grant dataset. For each promotional word, we calculated the percentage of grants that used the specific word and sorted the frequencies from high to low. We checked if the instances of these 139 words were used in a promotional way by identifying n-grams around each promotional word, and we discounted promotional words that were used as non-promotional. For example, "First" and "Intellectual" were used non-promotionally in "the first year" and "intellectual property."

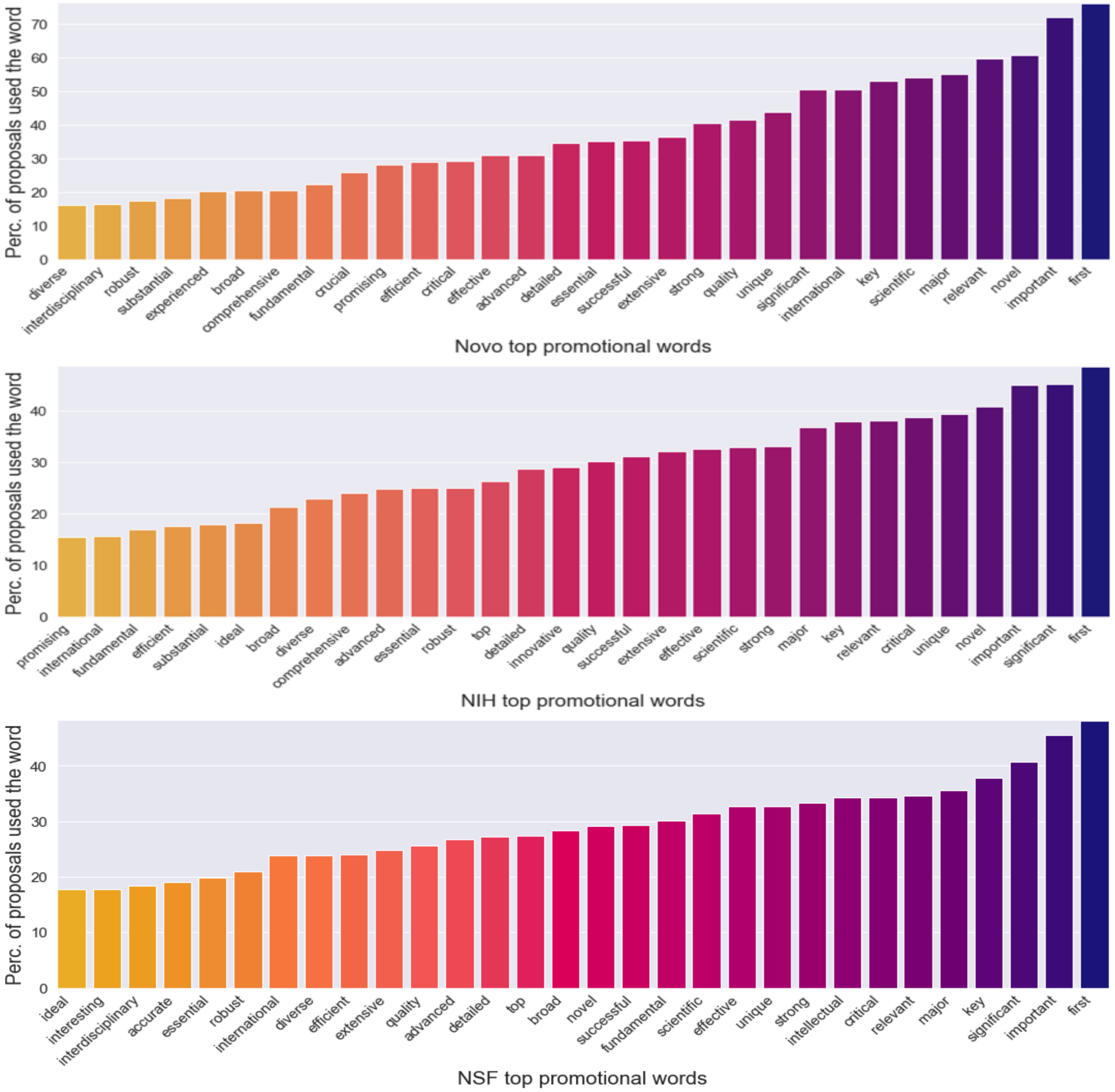

Figure S1. Promotional words ranked by the percentage of proposals that used a given word. Words such as "First" and "Important" are ranked the highest across all three datasets.

IV. Innovativeness Measure

Following prior research (2, 12-15), our study used the novelty measure at the paper level by calculating the novelty score for each journal combination based on prior studies the paper referenced. Specifically, for each journal pair in a given publication year, we compared its observed co-citation frequency in that year in the whole scientific literature with a null model of journal pairing distribution created by randomized citation networks, where citation links between papers were switched randomly with appropriate constraints. The constraint preserves the number of citations of each paper and the number of citations between year pairs. The null model is generated 10 times to produce a z-score for each journal pair for each publication year.

Journal pairs that appear more often than the expected value in the null model (positive z-scores) are considered conventional whereas those appear less than the expected value (negative z-scores) are considered novel. Each paper has a distribution of novelty scores with one score for each journal combination in its reference. We took the median of all negative z-scores of a paper (or grant in our case) and reverse coded this summary statistic as its innovativeness score per Kim, Cerigo, Jeong and Youn (16) such that a larger innovativeness score indicates a higher degree of innovativeness.

To make this measure work at the grant level, we used the reference section of each grant proposal to compute its innovativeness with the journal pair z-scores pre-computed based on papers and their citation links indexed in the Microsoft Academic Graph bibliographic database. We computed the innovativeness score for the NNF dataset but were unable to do so for the NIH and NSF proposals because they did not include intact references due to privacy considerations.

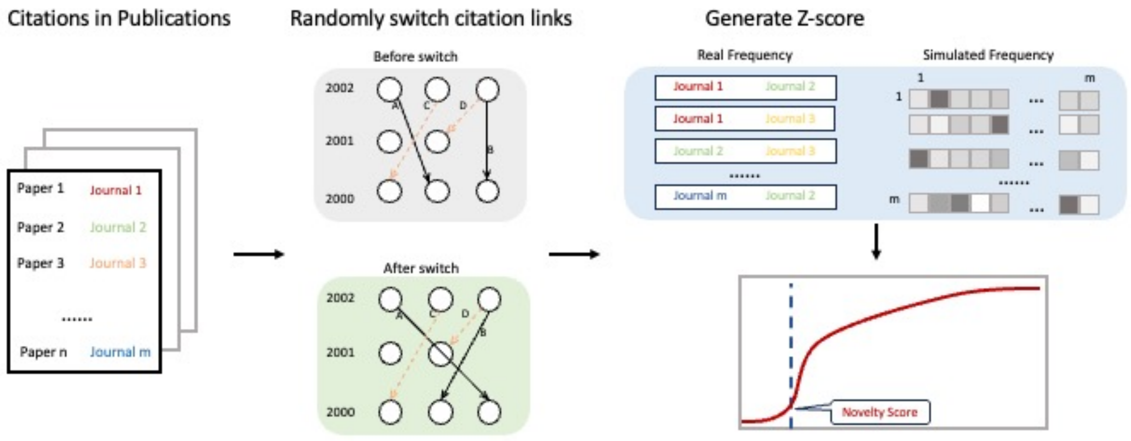

Figure S2. Illustration of the computation of novelty score for papers.

Overview

To measure the observed frequency of journal pairs in the grant's bibliography, we did the following. After having counted the observed yearly co-citation frequency of each journal pairing in the whole scientific literature (we used the Microsoft Academic Graph database), we computed the yearly frequency for each journal pairing that would have occurred by chance. The null model

randomly reassigns the citation links between papers using a variation of the Markov Chain Monte Carlo (MCMC) algorithm to randomly switch citations between all papers into a synthetic network (edges) with the same number of papers and citations as the empirical network. Note that this method preserves the detailed paper-level structure of the global citation network. Specifically, the number of citations to and from each paper is preserved, as are the dynamics of citation timing. Looking across ten randomized cases of the citation network, we generated a distribution of yearly co-citation frequencies for each journal pair. We can then calculate the z-score for each observed journal pair relative to what was expected by chance.

To measure a grant's level of innovativeness, we can now assign a z-score to each of the journal pairs in that grant's reference list. Each grant thus has a distribution of z-scores. To summarize the information in this distribution, we used the median of all negative z-scores of a grant as a summary statistic for the part of novel journal combinations that are relatively unusual compared to chance.

Illustrative Example of Methodology

To illustrate these procedures, we do the following to first pre-compute z-scores for all journal-pairs based on papers in the Microsoft Academic Graph dataset.

Step 1. Take references in the bibliography of a given paper published in 2000.
Step 2. Consider all pairwise combinations of the papers referenced in the bibliography of that paper. For example, paper 1 and paper 2 is a pair and paper 1 and paper 3 is a pair.
Step 3. Map the observed paper pairs into observed journal pairs for year 2000.
Step 4. Repeat steps (1)-(3) for every paper in the Microsoft Academic Graph.
Step 5. Count the yearly frequency of each observed journal pairing, which allows journal pair frequencies to vary over time.
Step 6. Create the null model. Repeat steps (1)-(5) to compute an expected frequency using the null model. Repeat this step 10 times.
Step 7. Compare the observed and expected frequency of journal pairs to compute a z-score for each journal pair on a yearly basis.

Now for a given grant, we do the following to calculate its innovativeness score:

Step 1. Take references in the bibliography of a grant titled "No-chemistry manufacture of nanocellulose for biocomposites," which was submitted in year 2000 and references 9 papers or 9 journals:

Materials Today
Cellulose
Biomacromolecules
Macromolecules
Biotechnology for Biofuels
Composites Science and Technology
Nature Communications
Applied and Environmental Microbiology
Nature Communications

Step 2. Get all 36 journal pairs (such as "*Cellulose – Nature Communications")*. Obtain a distribution of 36 z-scores for this grant, with one score for each journal pair in year 2000.

Step 3. To determine a grant's innovativeness score, we calculate the median of all z-scores that are below 0 (i.e., the median level of novel combinations that happen less than expected by chance) and reverse code it. The more the grant combines ideas in new and rare ways, the higher is its innovativeness score. In the case of grant, "No-chemistry manufacture of nanocellulose for biocomposites," the innovativeness score is 13.03, which is at the 70th percentile of innovativeness among all NNF grants.

V. Word Replacement Experiments

Neutral Non-promotional Synonyms of Promotional Words. We obtained synonyms for each promotional word from the Oxford Dictionary. We did two robustness checks on the synonyms. First, in a few cases, some synonyms are themselves a promotional word in our dictionary. For example, the word "critical" is a synonym for "crucial" according to the Oxford Dictionary, but "critical" is also a promotional word in our dictionary. We thus removed all synonym words that were also in the promotional word dictionary. Second, we employed a graduate student who was independent of the research team and who was majored in psychology with five years of work experience in university grant offices, to manually check whether each remaining synonym made sense in grant writing context. For example, the word "handsome" is a valid synonym for the promotional word "attractive" but is not used in scientific writing. Such synonyms were dropped from the synonym list to ensure that all synonyms used in our analysis were valid and were of non-promotional nature in the context of grant.

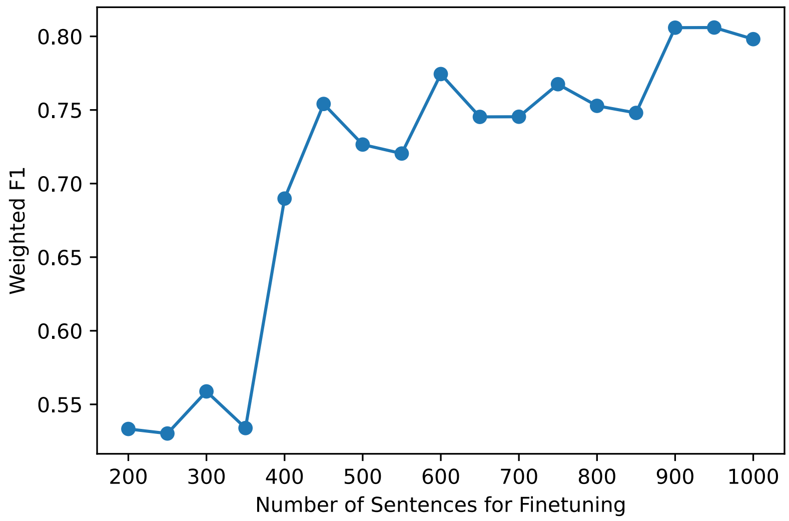

Figure S3. Cross-validated F1 scores of the SciBert model fine-tuned with different number of labeled sentences.

The non-promotional synonyms used for each promotional word are:

accessible: achievable, acquirable, approachable, attainable, available, comprehensible, fathomable, graspable, intelligible, obtainable, penetrable, procurable, reachable, realizable, securable, understandable, welcoming
accurate: authentic, close, correct, error-free, errorless, exact, fact-based, factual, fair, faithful, faultless, literal, precise, precision, realistic, reliable, right, specific, sure, truthful, unambiguous, unerring, valid, veracious
actionable: corrupt, culpable, delinquent, felonious, fraudulent, illegal, illegitimate, illicit, lawbreaking, nefarious, unlawful, villainous
advanced: futuristic, higher, higher-level, modern, new, newfangled, state-of-the-art
alarming: agitating, alerting, disconcerting, disquieting, distressing, disturbing, fearing, flustering, frightening, frighting, panicking, scary, shocking, terrifying, unease, upsetting
ambitious: arduous, aspiring, avid, challenging, committed, demanding, desirous, difficult, driven, eager, enterprising, exacting, formidable, go-ahead, go-getting, hankering, hoping, hungry, impatient, itching, onerous, pioneering, progressive, purposeful, pushy, stiff, strenuous, striving, zealous
ample: abundant, adequate, capacious, commodious, copious, enough, flush, generous, plentiful, profuse, roomy, sizeable, sufficient, teeming, wide
attractive: fascinating, fetching, inviting, pleasing, prepossessing, striking, stunning
biggest: consequential, far-reaching, high-priority, hugest, megalithic, mightiest, prodigious, robustest, sizeable, solidest, sturdiest, vastest
broad: across, all-embracing, approximate, broad-ranging, coarse, conspicuous, dirty, earthy, filthy, fuzzy, general, immeasurable, improper, inclusive, indecent, indecorous, indefinite, indelicate, loose, manifest, naughty, non-specific, noticeable, obvious, overall, overt, patent, plain, prominent, pronounced, racy, ribald, risqué, rough, rude, sizeable, sweeping, unfocused, unmistakable, unrefined, unspecific, unsubtle, vague, vulgar, wide, wide-ranging
careful: attentive, cautious, conscientious, deliberate, diligent, fastidious, hypervigilant, judicious, methodical, mindful, orderly, perfectionist, prudent, scrupulous, sedulous, sensible, vigilant, wary, watchful
cohesive: adhering
compelling: cogent, conclusive, credible, engrossing, fascinating, influential, persuasive, plausible, potent, profound, reasonable, reasoned, valid
comprehensive: all-inclusive, broad-based, complete, diversified, eclectic, inclusive, indiscriminate, wide
confident: assured, hopeful, optimistic, positive
considerable: appreciable, eminent, influential, much, noted, noteworthy, prominent, sizeable
creative: accomplished, experimental, genius, gifted, imaginative, innovational, innovatory, inventive, original
critical: analytic, analytical, commentative, criticizing, dangerous, deciding, decisive, disapproving, disparaging, evaluative, expository, grave, high-priority, negative, perilous, precarious, pre-eminent, reproving, risky, uncertain, unfavourable
crucial: deciding, decisive, determining, high-priority, mandatory, necessary, needed, pre-eminent, pressing, required, requisite
daunting: disconcerting, formidable, intimidating
dedicated: allocated, assigned, committed, devoted, faithful, resolute, staunch, steadfast, unwavering
deeper: absorbed, bass, deep-rooted, deep-seated, downwards, hidden, intenser, inwards, lower, low-pitched, obscurer, opaquer, profound, recondite, richer, riveted, steeped, wholehearted
desperate: dangerous, despairing, grave, perilous, precarious, pressing, risky, wanting
detailed: all-inclusive, complete, full
devastating: calamitous, cataclysmic, catastrophic, destructive, disastrous, distressing, incisive, ruinous, shattering, shocking, striking, stunning, terrible
dire: appalling, awful, distressing, drastic, dreadful, extreme, frightful, grave, grim, harrowing, horrible, ominous, portentous, pressing, shocking, terrible
dismal: abject, awful, bad, bleak, dark, desolate, dim, dingy, dispirited, doleful, downcast, drab, dreadful, dull, forlorn, glum, grim, lamentable, melancholy, pitiful, poor, sombre, terrible, woeful, wretched
diverse: manifold, multiple, sundry, various
durable: abiding, continuing, enduring, indestructible, lasting, long-lasting, long-term, persistent, persisting, resistant
easy: facile, painless, simple, unchallenging, uncomplicated, unconstrained, undemanding, undisturbed
effective: closing, concluding, constructive, effectual, end, functional, implicit, implied, operative, plausible, potent, practical, tacit, valid

efficacious: constructive, effectual, functional, potent
efficient: coherent, cost-effective, energy-efficient, energy-saving, fuel-efficient, labour-saving, logical, methodical, orderly, streamlined, structured, systematized
emerging: appearing, transpiring, unfolding
enormous: wide
essential: cardinal, characteristic, chief, compulsory, consequential, elemental, high-priority, inherent, innate, intrinsic, mandatory, necessary, needed, obligatory, pre-eminent, pressing, primary, principal, quintessential, required, requisite, rudimentary, underlying
exceptional: abnormal, atypical, excellent, prodigious, rare, singular, special, uncommon, unexpected, unusual
exciting: inspiring, invigorating, moving, rousing, stimulating
expansive: all-embracing, cross-disciplinary, inclusive, sweeping, wide, wide-ranging
experienced: accomplished, adept, adroit, consummate, expert
extensive: complete, immeasurable, large-scale, profound, sizeable
fastest: quickest, rapidest, speediest, swiftest
first: basal, beginning, best, chief, earliest, elemental, firstly, foremost, foundation, highest, initial, initiatory, introductory, launching, leading, main, novelty, opening, original, primary, prime, principal, rather, rudimentary, sooner, superlative, topmost, underlying, uppermost, utmost
fundamental: basal, deciding, decisive, elemental, foundational, high-priority, pre-eminent, root, rudimentary, underlying
greatest: accomplished, adeptest, adroitest, amplest, appreciable, broadest, complete, dominant, eminent, exceedingly, extraordinarily, extremely, immensely, imposing, impressive, influential, potent, pre-eminent, proficient, prominent, pronounced, redoubtable, remarkably, salient, sizeable, strongest, tremendously
huge: prodigious
ideal: archetypal, complete, conceptual, consummate, exemplary, faultless, flawless, impracticable, model, notional, philosophical, quintessential, supreme, theoretical, unachievable, unattainable, unfeasible
immediate: close, closest, current, expeditious, instant, instantaneous, near, nearest, on-the-spot, present, primary, prompt, quick, rapid, recent, speedy, swift
immense: prodigious
imperative: commanding, exigent, necessary, peremptory, pressing
important: cardinal, chief, consequential, dominant, far-reaching, foremost, formidable, high-level, influential, main, necessary, overriding, predominant, prime, principal, salient, supreme, top-level, useful, valuable, valued
incredible: imposing, impressive, prodigious, staggering
indispensable: compulsory, consequential, high-priority, mandatory, necessary, needed, obligatory, pre-eminent, pressing, required, requisite
innovative: avant-garde, experimental, forward-looking, fresh, futuristic, groundbreaking, innovational, innovatory, inventive, modern, new, original, pioneering, progressive, state-of-the-art, trailblazing, unconventional, unorthodox, unusual
intellectual: cognitive, mental, scholarly
interdisciplinary: cross-disciplinary
interesting: engrossing, fascinating
international: global, intercontinental, worldwide
intriguing: drawing, fascinating
intuitive: instinctive, instinctual, intuitional
invaluable: irreplaceable
key: central, chief, decisive, dominant, leading, main, prime, principal, salient
largest: abundant, broadest, bulkyist, far-reaching, heftiest, hugest, large-scale, macroscale, sizeable, solidest, sufficient, tallest, vastest, wide-ranging, wide-reaching, widest
latest: contemporary, current, fresh, modern, modernistic, newest, present-day, state-of-the-art
longstanding: established, long-established
long-standing: established, long-established
major: best, capital, cardinal, chief, difficult, eminent, foremost, leading, main, pre-eminent, prime, principal, supreme, sweeping, top-tier, uppermost, utmost
massive: bulky, prodigious, staggering
meaningful: consequential, expressive, pithy, pointed, purposeful, sincere, valid, worthwhile
motivated: encouraged, excited, galvanized, inspired, roused, spurred, stimulated
multidisciplinary: complete, profound

myriad: countless, immeasurable, innumerable, legion, limitless, manifold, many, multifarious, multiple, multitudinous, numberless, numerous, several, sundry, unlimited, unnumbered, various
notable: acclaimed, celebrated, consequential, conspicuous, eminent, esteemed, glaring, honoured, impressive, influential, marked, memorable, noted, noteworthy, obvious, particular, pre-eminent, prominent, pronounced, rare, respected, signal, special, striking, uncommon, unusual
novel: avant-garde, different, fresh, futuristic, groundbreaking, imaginative, innovational, innovatory, inventive, modern, new, original, pioneering, state-of-the-art, trailblazing, unconventional, unorthodox, unusual
outstanding: celebrated, consequential, detectable, discernible, distinctive, due, eminent, excellent, formidable, historic, impressive, incomplete, left, memorable, neglected, noteworthy, noticeable, observable, omitted, ongoing, overdue, pending, perceivable, perceptible, pre-eminent, remaining, signal, special, striking, superlative, undischarged, undone, unfinished, unsettled, visible, vivid
overwhelming: formidable, inordinate, prodigious, profound, profuse, shattering, staggering, sweeping
paramount: incomparable, inimitable, matchless, peerless, unequalled, unexcelled, unmatched, unsurpassed
pivotal: central, deciding, decisive, determining, focal
powerful: cogent, commanding, consuming, dominant, dynamic, formidable, impressive, influential, persuasive, potent, redoubtable, solid, striking, vigorous
premier: best, chief, choice, elite, excellent, foremost, head, highest, high-grade, leading, main, peerless, pre-eminent, primary, prime, principal, quality, select, superior, superlative, top-class, top-grade, top-quality, top-ranking, top-tier, unexcelled, unsurpassed
prestigious: acclaimed, celebrated, eminent, esteemed, estimable, exalted, honoured, imposing, impressive, influential, leading, prominent, reputable, respected
productive: constructive, fertile, gainful, gratifying, helpful, high-yielding, inventive, profitable, rewarding, useful, valuable, worthwhile
promising: encouraging, favourable, hopeful, optimistic, positive, propitious, reassuring, rising, up-and-coming
qualified: bounded, cautious, certificated, certified, chartered, circumscribed, conditional, contingent, equivocal, licensed, limited, restricted, tentative
ready: achievable, acquirable, apt, arranged, attainable, available, completed, disposed, done, equipped, fast, finished, given, inclined, likely, minded, organized, predisposed, prepared, primed, procurable, prompt, prone, quick, rapid, realizable, securable, set, speedy, swift, unhesitating, willing
relevant: admissible, applicable, apposite, apropos, germane, pertinent
remarkable: prodigious, stunning
renowned: acclaimed, celebrated, eminent, esteemed, pre-eminent, prominent
revolutionary: avant-garde, complete, different, disruptive, entire, factious, far-reaching, fresh, futuristic, groundbreaking, imaginative, innovational, innovatory, inventive, modern, new, original, pioneering, profound, seditious, state-of-the-art, subversive, sweeping, trailblazing, unconventional, unorthodox, unusual, wide-ranging
rich: abundant, arable, copious, fertile, full, generous, plentiful, profitable, profit-making, profuse, propertied, prosperous, vivid
rigorous: attentive, austere, bad, bleak, conscientious, correct, cruel, demanding, despotic, diligent, exact, exacting, extreme, harsh, mathematical, methodical, particular, perfectionist, precise, relentless, rigid, scrupulous, stringent, ultra-careful, uncompromising, unsparing
robust: long-lasting, resilient, vigorous
safer: attentive, benign, cautious, defended, harmless, impregnable, innocuous, invulnerable, low-risk, mild, non-irritant, non-poisonous, non-toxic, prudent, risk-free, riskless, sheltered, shielded, unassailable, undamaged, unharmed, unhurt, uninjured, unscathed
scientific: controlled, exact, mathematical, methodical, ordered, orderly, organized, precise, regulated, technical, technological
senior: chief, elder, higher-ranking, highest-ranking, high-status, older, superior
significant: consequential, expressive, indicative, informative, knowing, noteworthy, pithy, purposeful, revealing, valid
skilled: able, accomplished, adept, adroit, capable, competent, deft, dexterous, expert, gifted, practised, proficient, smart, trained, versed
sophisticated: innovatory, trailblazing, worldly
stark: arid, austere, bare, barren, bleak, blunt, complete, crisp, desolate, distinct, empty, evident, grim, harsh, obvious, outright, plain, positive, pure, sharp, sheer, simple, sombre, striking, unadorned, uncomfortable, undecorated, unembellished, unvarnished, vacant, vigorous
stellar: singular, staggering, stunning

strategic: calculated, deliberate, planned
strong: able, accomplished, adept, adroit, biting, capable, cogent, concentrated, deep-seated, drastic, dynamic, eager, earnest, extreme, formidable, full, glaring, heady, healthy, hearty, impenetrable, impregnable, indestructible, inviolable, loud, plausible, potent, proficient, profound, pungent, redoubtable, resolute, sharp, solid, staunch, steadfast, stiff, striking, tenacious, thriving, unassailable, undiluted, valid, vehement, vigorous, vivid, zealous
substantial: appreciable, decent, long-lasting, marked, profitable, profit-making, prosperous, real, sizeable, solid, useful, valuable, worthwhile
successful: burgeoning, flourishing, gainful, lucrative, moneymaking, profitable, profit-making, prosperous, solvent, thriving, triumphant, victorious
surprising: agape, confounding, jolted, jolting, nonplus, shocked, shocking, stagger, staggered, stunning, unanticipated, unexpected, unforeseen, unpredicted
sustainable: acceptable, dependable, just, justified, legitimate, reasonable, reliable, sensible, sure, trustworthy, valid
synergy: accord, association, collaboration, compromise, concord, concurrence, coopetition, coordination, liaison, partnership, teamwork, understanding, unity
systematic: coherent, consistent, fastidious, formal, logical, methodical, orderly, organized, planned, practical, regular, routine, standard, standardized, structured, systematized
tailored: adapted, adjusted, altered, attuned, changed, converted, fitted, geared, modified, moulded, reshaped, shaped, suited, tuned
talented: able, accomplished, adept, adroit, apt, capable, competent, consummate, deft, dexterous, expert, gifted, polished, proficient
tangible: appreciable, concrete, corporeal, definite, discernible, distinct, evident, indisputable, intelligible, manifest, measurable, obvious, palpable, perceptible, physical, positive, real, solid, striking, tactile, touchable, undoubted, unmistakable, verifiable
timely: appropriate, apt, convenient, expedient, felicitous, fitting, opportune, prompt, punctual, suitable
top: best, chief, choice, commanding, elite, excellent, foremost, high, highest, high-grade, leading, main, maximal, maximum, peerless, pre-eminent, prime, principal, quality, select, superior, superlative, top-class, top-grade, topmost, top-quality, top-tier, unexcelled, unsurpassed, upmost, upper, uppermost, utmost
transformative: alternative, changable, converted, metamorphosing, modifiable, mutatative, transfigurative, transmutative
tremendous: excellent, prodigious
ultimate: best, central, concluding, conclusive, definitive, elemental, end, endmost, eventual, furthest, highest, last, optimum, primary, prime, quintessential, superlative, supreme, terminal, topmost, unrivalled, unsurpassed, utmost
unanswered: disputed, pending, undecided, undetermined, unresolved, unsettled
unique: distinctive, eccentric, idiosyncratic, individual, isolated, noteworthy, particular, peculiar, quirky, signal, singular, special, specific
unparalleled: rare, singular, unequalled
unprecedented: unequalled, unmatched, unrivalled
urgent: dogged, drastic, emergency, extreme, grave, high-priority, obstinate, persistent, pressing, resolute, tenacious, top-priority, unrelenting
user-friendly: articulate, coherent, comprehensible, crystalline, intelligible, lucid, understandable
vast: immeasurable, limitless, prodigious, wide
vibrant: animated, blaring, dynamic, echoing, effervescent, full, pulsating, quaking, quavering, quavery, quivering, reverberant, reverberating, stimulating, strident, striking, vigorous, vivid
vital: active, animated, consequential, dynamic, high-priority, life-preserving, life-sustaining, mandatory, necessary, needed, pre-eminent, pressing, required, requisite, vigorous

References

1. B. Uzzi, S. Mukherjee, M. Stringer, B. Jones, Atypical combinations and scientific impact. *Science* **342**, 468-472 (2013).
2. Y. Yang, T. Y. Tian, T. K. Woodruff, B. F. Jones, B. Uzzi, Gender-diverse teams produce more novel and higher-impact scientific ideas. *Proceedings of the National Academy of Sciences* **119**, e2200841119 (2022).
3. M. Brysbaert, A. B. Warriner, V. Kuperman, Concreteness ratings for 40 thousand generally known English word lemmas. *Behavior research methods* **46**, 904-911 (2014).
4. J. N. Farr, J. J. Jenkins, D. G. Paterson, Simplification of Flesch reading ease formula. *Journal of applied psychology* **35**, 333 (1951).
5. N. Millar, B. Batalo, B. Budgell, Trends in the use of promotional language (hype) in abstracts of successful national institutes of health grant applications, 1985-2020. *JAMA Network Open* **5**, e2228676-e2228676 (2022).
6. N. Millar, F. Salager-Meyer, B. Budgell, "It is important to reinforce the importance of…":'Hype'in reports of randomized controlled trials. *English for Specific Purposes* **54**, 139-151 (2019).
7. D. T. Campbell, D. W. Fiske, Convergent and discriminant validation by the multitrait-multimethod matrix. *Psychological bulletin* **56**, 81 (1959).
8. S. Mohammad, Obtaining reliable human ratings of valence, arousal, and dominance for 20,000 English words. *Proceedings of the 56th Annual Meeting of the Association for Computational Linguistics*, 174-184 (2018).
9. F. M. Citron, M. A. Gray, H. D. Critchley, B. S. Weekes, E. C. Ferstl, Emotional valence and arousal affect reading in an interactive way: neuroimaging evidence for an approach-withdrawal framework. *Neuropsychologia* **56**, 79-89 (2014).
10. A. E. Raftery, Bayesian model selection in social research. *Sociological methodology*, 111-163 (1995).
11. K. Börner *et al.*, Design and update of a classification system: The UCSD map of science. *PloS one* **7**, e39464 (2012).
12. L. Wu, D. Wang, J. A. Evans, Large teams develop and small teams disrupt science and technology. *Nature* **566**, 378 (2019).
13. M. Park, E. Leahey, R. J. Funk, Papers and patents are becoming less disruptive over time. *Nature* **613**, 138-144 (2023).
14. E. Leahey, C. M. Beckman, T. L. Stanko, Prominent but less productive: The impact of interdisciplinarity on scientists' research. *Administrative Science Quarterly* **62**, 105-139 (2017).
15. H. Youn, D. Strumsky, L. M. Bettencourt, J. Lobo, Invention as a combinatorial process: evidence from US patents. *Journal of the Royal Society interface* **12**, 20150272 (2015).
16. D. Kim, D. B. Cerigo, H. Jeong, H. Youn, Technological novelty profile and invention's future impact. *EPJ Data Science* **5**, 1-15 (2016).
